# Response of Cisplatin Resistant Skov-3 Cells to [Pt(*O,O′*-Acac)(γ-Acac)(DMS)] Treatment Revealed by a Metabolomic ^1^H-NMR Study

**DOI:** 10.3390/molecules23092301

**Published:** 2018-09-09

**Authors:** Federica De Castro, Michele Benedetti, Giovanna Antonaci, Laura Del Coco, Sandra Angelica De Pascali, Antonella Muscella, Santo Marsigliante, Francesco Paolo Fanizzi

**Affiliations:** Dipartimento di Scienze e Tecnologie Biologiche ed Ambientali, Università del Salento, Via Monteroni, I-73100 Lecce, Italy; federica.decastro@unisalento.it (F.D.C.); giovanna.antonaci@unisalento.it (G.A.); laura.delcoco@unisalento.it (L.D.C.); sandepa7@gmail.com (S.A.D.P.); antonella.muscella@unisalento.it (A.M.); santo.marsigliante@unisalento.it (S.M.)

**Keywords:** cisplatin, platinum based drugs, [Pt(*O*,*O′*-acac)(γ-acac)(DMS)], Ptac2S, Epithelial Ovarian Carcinoma, SKOV-3 cells, ^1^H-NMR metabolomics

## Abstract

The novel [Pt(*O*,*O′*-acac)(γ-acac)(DMS)], Ptac2S, Pt(II) complex has recently gained increasing attention as a potential anticancer agent for its pharmacological activity shown in different tumor cell lines, studied both in vitro and in vivo. The mechanism of action of Ptac2S, operating on non-genomic targets, is known to be very different from that of *cis*-[PtCl_2_(NH_3_)_2_], cisplatin, targeting nucleic acids. In this work, we evaluated the cytotoxicity of Ptac2S on the cisplatin resistant Epithelial Ovarian Carcinoma (EOC), SKOV-3 cells, by the MTT assay. A ^1^H-NMR metabolomic approach coupled with multivariate statistical analysis was used for the first time for Ptac2S to figure out the biological mechanisms of action of the complex. The metabolic variations of intracellular metabolites and the composition of the corresponding extracellular culture media were compared to those of cisplatin (cells were treated at the IC_50_ doses of both drugs). The reported comparative metabolomic analysis revealed a very different metabolic profile between Ptac2S and cisplatin treated samples, thus confirming the different mechanism of action of Ptac2S also in the Epithelial Ovarian Carcinoma (EOC), SKOV-3 cells line. In particular, higher levels of pyruvate were observed in Ptac2S treated, with respect to cisplatin treated, cells (in both aqueous and culture media). In addition, a very different lipid expression resulted after the exposure to the two drugs (Ptac2S and cisplatin). These results suggest a possible explanation for the Ptac2S ability to circumvent cisplatin resistance in SKOV-3 cells.

## 1. Introduction

The development of new approaches to investigate tumor responses to chemotherapy is a critical point for the improvement of clinical outcomes in cancer treatment [1,2]. Over several of the last decades, metabolomic studies have provided a significant contribution to this field. In the 1920s Otto Warburg observed that cancer cells have an abnormally high rate of aerobic glycolysis and convert pyruvate (the glycolysis end product) into lactate, due to the impaired mitochondrial function, the well-known “Warburg effect” [3,4,5]. In addition to that, cancer cells exhibit other unique metabolic characteristics such as increased fatty acid synthesis and glutamine metabolism [5].

The metabolic alterations in cancer cells, together with the variations of metabolic pathways caused by drug stimuli, have generated a great interest toward the application of metabolomics technologies in oncology [6,7,8,9]. Recently, metabolomics has been greatly used to analyze tissue or bio-fluid samples in order to discover diagnostic cancer biomarkers as well as to get a better understanding of the metabolic pathways alteration in cancer cells [10,11,12]. The study of metabolic profiles represents a helpful technique for drug response assessment, action mechanism investigation, and drug resistance analysis [13].

In this regard, NMR-based metabolomics of cells, tissue, and biological fluids demonstrated as a valid and extremely sensitive approach to detect metabolic effects under drug exposure. Over the last years [14,15,16,17,18,19] the effects of a number of different compounds have been investigated by means of this technique, including many DNA alkylating agents and cisplatin itself [20,21,22,23,24,25].

**Figure 1:**
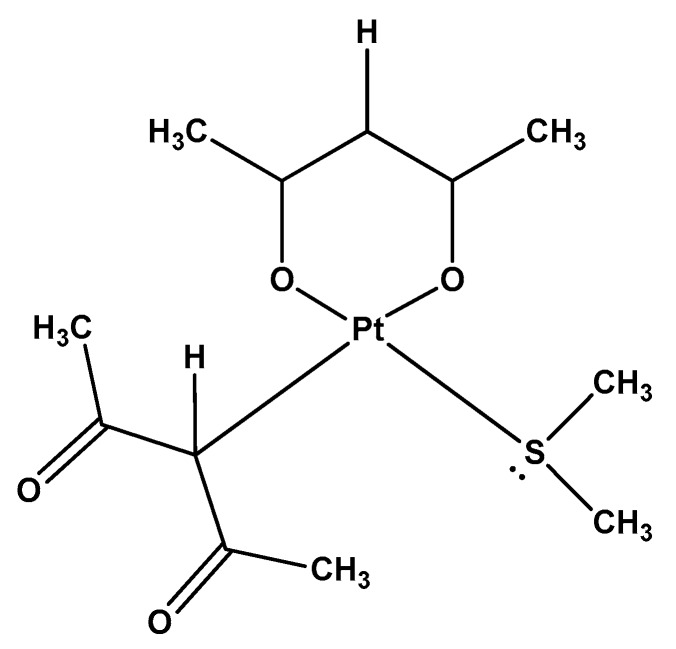
Chemical structure of [Pt(*O*,*O′*-acac)(γ-acac)(DMS)], Ptac2S.

Recently, our group reported the synthesis of a new Pt(II) compound, [Pt(*O,O′*-acac)(γ-acac)(DMS)], Ptac2S, (Figure 1) which has shown notable biological activities, both in vitro and in vivo [26,27,28,29]. The interactions of Ptac2S with non-genomic targets, which has been demonstrated in previous studies [30,31], revealed a mechanism of action of this compound very different from cisplatin, although not completely clarified yet. It is well known that cisplatin can cross the cell membrane by passive diffusion [32,33,34], or using selected ion channels [35]. Once inside the cytosol, aquation processes activate the drug, increasing its reactivity toward DNA (essentially at the purines N7 electron donors, which are considered the main pharmacological targets of cisplatin) [32,33,34,35,36,37,38,39,40,41,42,43,44,45].

On the other hand, the alternative action mechanism of Ptac2S (essentially cytosolic) might be responsible for the ability of this potential anticancer agent to overcome the drug resistance induction, one of the principal causes of cisplatin-based tumor treatment failure [46]. Indeed, Ptac2S has shown a higher in vitro and in vivo pharmacological activity and tolerability than cisplatin [47,48,49], making it an attractive alternative to cisplatin for the treatment of different tumor.

In this paper, we describe the application of a ^1^H-NMR-based metabolomic approach to evaluate the pharmacological activity of Ptac2S on cisplatin resistant Epithelial Ovarian Carcinoma (EOC), SKOV-3 cell line. The current therapy against EOC is a combined chemotherapy, which includes taxanes and platinating agents [50,51,52]. Despite early diagnosis and treatment, the tumor often develops into an aggressive and resistant form that leads patients to death [53]. Indeed, EOC nowadays represents one of the principal causes of death among women with an overall median survival of 5 years after diagnosis [54]. For this reason, the development of a new therapy able to overcome the often occurring resistance phenomena is highly desired. In light of the activity already shown on some cisplatin resistant carcinoma cells [28], we decided to investigate the effects of Ptac2S on SKOV-3 also by ^1^H-NMR based metabolomics. In order to probe the responses of SKOV-3 cells to Ptac2S’s exposure, multivariate spectroscopic data of both intracellular and extracellular medium SKOV-3 extracts following 24 h of Ptac2S treatment, at IC_50_ dose, were analyzed using chemo-metric and pattern recognition techniques, (Principal Component Analysis, PCA, and Orthogonal Partial Least Squares Discriminant Analysis, OPLS-DA). The variations in metabolic profiles were compared to those of untreated and cisplatin treated (at the IC_50_ dose) SKOV-3 cells in order to get more information about the mechanism of action of the considered complex and the possible pathways involved in cisplatin resistance phenomena.

## 2. Results and Discussion

### 2.1. *In Vitro* cytotoxicity of Ptac2S Complex in SKOV-3 Cells

**Figure 2:**
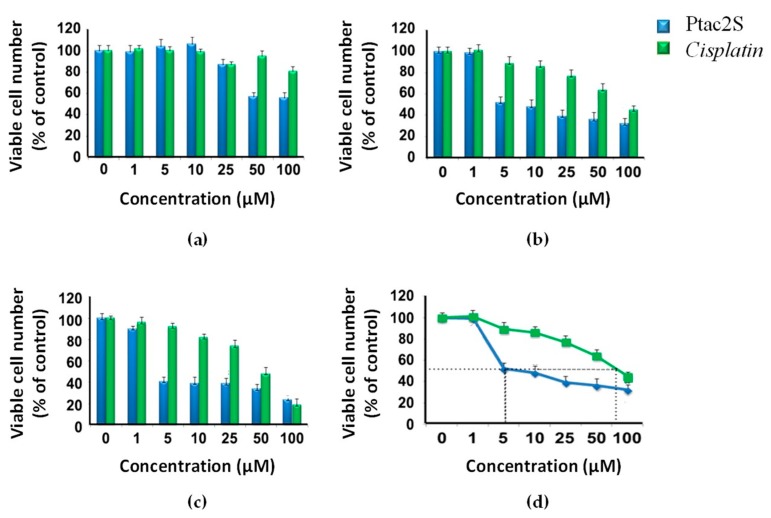
Evaluation of the cytotoxicity of Ptac2S on SKOV-3 cells in comparison with *cisplatin*. The SKOV-3 cells were treated with increasing concentrations of Ptac2S or *cisplatin* by determining the viable cell number after 12 h (**a**), 24 h (**b**), 48 h (**c**). Panel (**d**) shows the IC_50_ of Ptac2S and *cisplatin* after 24 h.

The MTT cytotoxicity assay was used to evaluate the effect of Ptac2S treatment on the SKOV-3 cells viability. Cell viability was compared to cisplatin treated and untreated SKOV-3 cells (in the 1–100 μM concentration range and 12–48 h time intervals), Figure 2. The exposure of SKOV-3 cells to Ptac2S and cisplatin at the concentration ranging from 1–100 μM resulted, as expected, in a dose-dependent inhibition of cell survival. Interestingly, significantly toxic effects due to Ptac2S, at dosages of 5 μM at 24 h, were found, Figure 2. The IC_50_ values (at 24 h) of Ptac2S and cisplatin calculation showed a Ptac2S cytotoxicity 14-fold higher than that observed for cisplatin, in detail equal to 6.2 µM ± 2.8 µM for Ptac2S and 87.5 ± 3.5 µM for cisplatin (last value confirms the intrinsic cisplatin resistance of SKOV-3 cells, as reported by the vendor, see Materials and Methods).

For the interesting Ptac2S cytotoxicity on this cisplatin resistant cell line, it is important to check the changes of cellular metabolome and composition of culture media to monitor the cytotoxicity of SKOV-3 cells in response to the exposure of Ptac2S.

### 2.2. Metabolic Alterations in SKOV-3 Cells Induced by Ptac2S Treatment

Cancer cells show an intrinsic metabolism which is quite different from that of healthy cells. For this reason, the analysis of cells metabolome represents a useful tool providing a detailed description of the tumor response to antitumor drugs [14,55,56,57,58]. In this work, we studied the metabolic alterations induced by Ptac2S treatment on SKOV-3 cells. A recognized NMR-based metabolomic approach was used. In order to get more detailed information about the mechanism of action of Ptac2S, metabolic profiles of untreated SKOV-3 cells were compared with that of Ptac2S and cisplatin treated SKOV-3 cells. To underline and better evidence the metabolic differences induced by both complexes the same experimental conditions, IC_50_ doses and sampling times were used. A multivariate data analysis was performed on the acquired ^1^H-NMR spectra of both aqueous (n = 27) and lipidic cell extracts (n = 27), at the considered (6, 12, and 24 h) time intervals, as well as in order to confirm direct spectroscopic NMR observations and identify the most statistically relevant changes.

#### 2.2.1. Metabolomic Studies of the Aqueous Skov-3 Cells Extracts.

**Figure 3:**
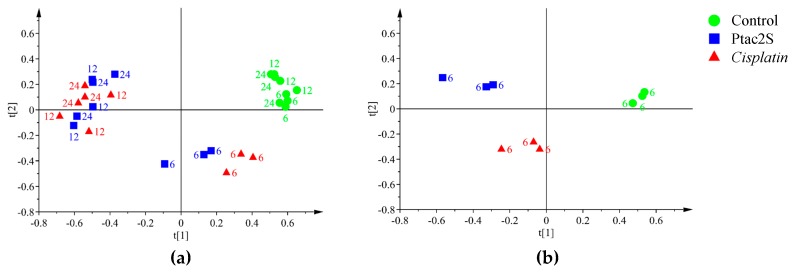
PCA score scatter plot of the 600 MHz ^1^H-CPMG-NMR spectra (Pareto scaled) obtained from aqueous extracts of Ptac2S and cisplatin treated SKOV-3 cells in comparison with the untreated control samples. The indicated labels are referred to the culture times. (**a**) PCA t[1]/t[2] (four components give R^2^X = 0.862, Q^2^ = 0.79). (**b**) PCA t[1]/t[2] scores scatter plot (two components give R^2^X = 0.865, Q^2^ = 0.754).

In order to analyze the intrinsic variation in the ^1^H-CPMG-NMR dataset, in the three treatment conditions (Ptac2S, cisplatin and untreated controls), the unsupervised pattern recognition method PCA (principal component analysis) was initially performed, Figure 3a.

In the PCA model, four components explained 86.2% of total variance, describing the samples distribution in model. The results showed a time dependent class separation between controls (untreated cells) and treated cells (either with Ptac2S and cisplatin). Specific clustering was observed for the controls (all sampling times), the shorter (6 h), and the longer time treated (12–24 h) samples. This separation resulted clear along the t[1] component, for the treated with respect to untreated samples and along the t[2] component for controls and long with respect to short times treated samples, Figure 3a. Interestingly, observing the treated samples (either with Ptac2S or cisplatin), the model indicated that the most relevant metabolic changes occurred at 6 h, Figure 3b.

**Figure 4:**
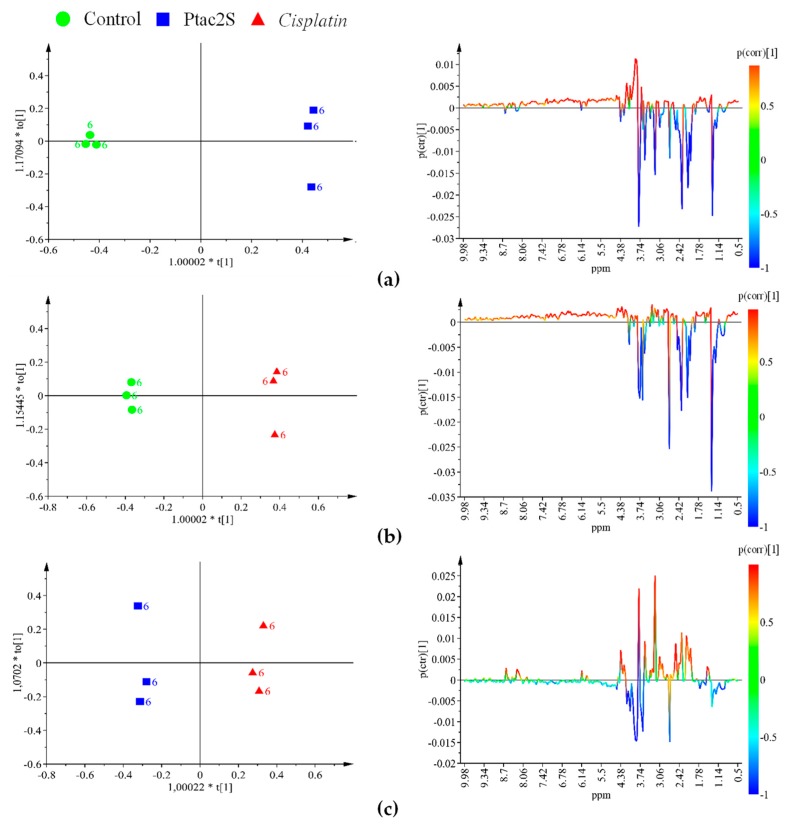
OPLS-DA score plot (**left** panel) and corresponding coefficient plot (**right** panel) of the 600 MHz ^1^H-CPMG-NMR spectra (Pareto scaled) of SKOV-3 cells aqueous extracts obtained from different pairwise groups (controls, Ptac2S and cisplatin) at 6 h. (**a**) OPLS-DA t[1]/t[2] scores scatter plot (two components, 1 predictive + 1 orthogonal, give R^2^X = 0.911, R^2^Y = 0.999, Q^2^ = 0.988). (**b**) OPLS-DA t[1]/t[2] scores scatter plot (two components, 1 predictive + 1 orthogonal give R^2^X = 0.886, R^2^Y = 0.999, Q^2^ = 0.984). (**c**) OPLS-DA t[1]/t[2] scores scatter plot (two components, 1 predictive + 1 orthogonal give R^2^X = 0.875, R^2^Y = 0.995, Q^2^ = 0.984).

**Figure 5:**
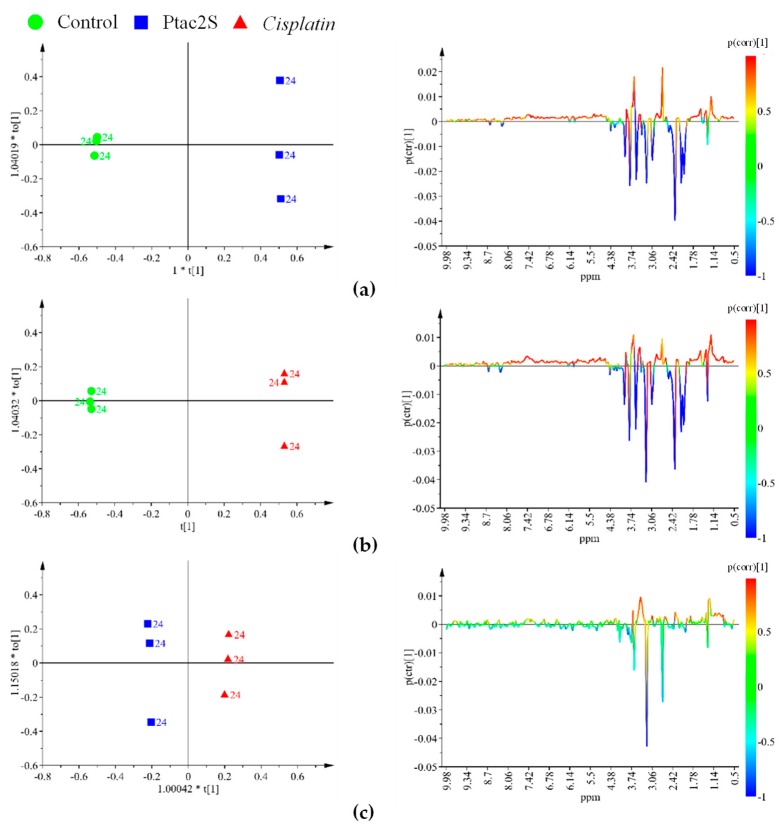
OPLS-DA score plot (**left** panel) and corresponding coefficient plot (**right** panel) of the 600 MHz ^1^H-CPMG-NMR spectra (Pareto scaled) of SKOV-3 cells aqueous extracts obtained from different pairwise groups (controls, Ptac2S, and cisplatin) at 24 h. (**a**) OPLS-DA t[1]/t[2] scores scatter plot (three components, 1 predictive + 2 orthogonal give R^2^X = 0.926, R^2^Y = 1, Q^2^ = 0.998). (**b**) OPLS-DA t[1]/t[2] scores scatter plot (three components, 1 predictive + 2 orthogonal give R^2^X = 0.931, R^2^Y = 1, Q^2^ = 0.999). (**c**) OPLS-DA t[1]/t[2] scores scatter plot (three components, 1 predictive + 2 orthogonal give R^2^X = 0.745, R^2^Y = 0.998, Q^2^ = 0.899).

With the aim to optimize the occurring metabolites’ differences, the samples corresponding to the three used conditions (Ptac2S or cisplatin treated and controls) were further analyzed by a supervised method (OPLS-DA, orthogonal partial least squares discriminant analysis) after 6 and 24 h treatment. The corresponding score and related S-line plots are reported in Figure 4 and Figure 5.

The OPLS-DA score plots and quality model parameters clearly indicated a good possibility to differentiate pairwise all the samples for all the used conditions, at 6 and 24 h (Figure 4 and Figure 5). A total of 24 metabolites resulted significantly responsible for the observed class separation and their related variations together with ^1^H-NMR relevant chemical shifts are reported in Table 1.

Ptac2S, at the early stage of exposure (6 h) gave interesting metabolic variations with respect to both controls and cisplatin. A decrease in the relative content of alanine, creatine, dimethylamine, glycine, glutamine, glutamate, phosphocholine (PC), succinate, AMP, taurine, as well as an increase in serine was observed in the Ptac2S with respect to the controls and cisplatin treated samples. On the other hand, in the Ptac2S treated samples, the levels of acetate, lactate, isoleucine, leucine, and valine were found lower and a little higher with respect to the controls and the cisplatin treated group, respectively. Furthermore, at 6 h, comparison of cisplatin with respect to Ptac2S treated and controls samples showed increased levels of PC and creatine. Moreover, in the cisplatin treated group, the levels of succinate, taurine, alanine, and AMP resulted strictly comparable to that of controls (Figure 4).

At the longer time point (24 h), in the Ptac2S treated samples, a considerable down regulation of acetate, alanine, AMP, creatine, glycine, glutamine, glutamate, lactate, myo-inositol, PC, succinate, taurine, with respect to the controls, was observed. Differently, the comparison of Ptac2S with respect to cisplatin treated samples, at the same time of exposure (24 h), resulted in higher levels of choline (Cho), glycerol, and pyruvate. Interestingly, no traces of pyruvate and very low levels of Cho were found in cisplatin treated cells after 24 h (Figure 5).

**Figure 6:**
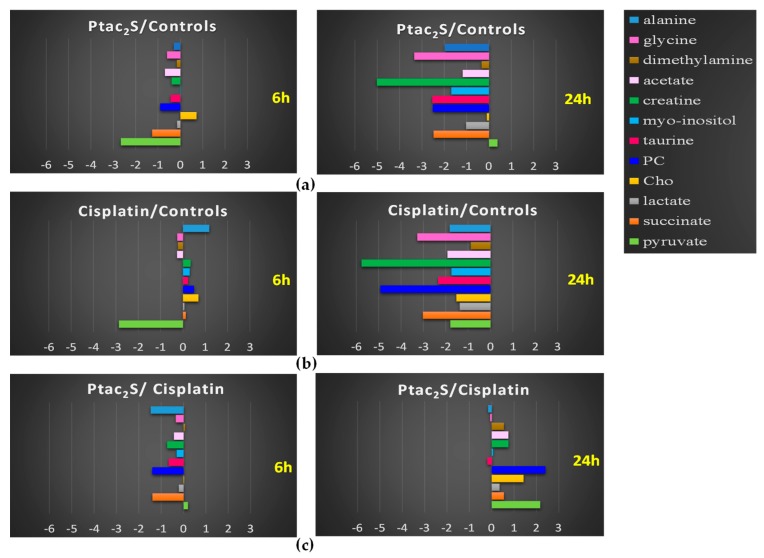
Relevant discriminant metabolites comparison of SKOV-3 cells aqueous extracts obtained from different pairwise groups (controls, Ptac2S, and cisplatin). The values of −Log2(FC) and the *p*-values are provided (Student’s *t* test, *p*-value < 0.05). (**a**,**b**) Metabolites with −Log2 (FC) negative values have lower concentration with respect to those of the 6 h (left panel) and 24 h (right panel) of the control, while −Log2 (FC) positive values have higher concentration with respect to those of the 6 h (left panel) and 24 h (right panel) of the control. (**c**) Metabolites with −Log2 (FC) negative values have lower concentration with respect to those of the 6 h (left panel) and 24 h (right panel) of cisplatin, while −Log2 (FC) positive values have higher concentration with respect to those of the 6 h (left panel) and 24 h (right panel) of cisplatin.

In order to evaluate the time course of the main metabolic variations between Ptac2S and cisplatin, a targeted analysis was performed. The spectral integration of relevant metabolites observed in the previously described models was carried out whenever possible. Fold change (FC) ratio (log2) of the normalized median intensity for the distinctive signals in the spectra corresponding to the previous discussed conditions was calculated. The *p*-values from Student’s test were also evaluated. Pairwise comparisons of the fold change variation for the identified metabolites are reported in Figure 6. The trend detected by Log2 fold change (FC) analysis could be also confirmed by direct comparison of representative sample spectra.

As shown above, the Ptac2S treated samples, at the early stage of exposure (6 h) revealed a statistically significant lower concentration of lactate, succinate (a Krebs cycle intermediate), and glutamine with respect to the controls and cisplatin treated samples. The well-known Warburg effect established that the metabolic reprogramming of tumor cells modifies cellular metabolic fluxes and enhanced glycolysis. The enhanced glycolysis leads to the enhanced conversion of pyruvate (the end-product of glycolysis) into lactate by lactate dehydrogenase [3,4]. Lactate is excreted from cells, whereas glutamine is used as a carbon atoms source for the Krebs cycle [59].

In the Ptac2S treated samples, at 6 h, the lowering of lactate and glutamine levels and the parallel decrease of succinate probably indicated the modulation of Krebs cycle activity induced by Ptac2S [59]. The levels of pyruvate in Ptac2S treated samples, at 6 h, are strictly comparable with that of controls and cisplatin. Interestingly, differently from controls and cisplatin, in Ptac2S treated samples the levels of pyruvate are not supported by the increase of lactate, but rather by its decrease. This last condition suggests the possible modulation of the activity of lactate dehydrogenase, the enzyme responsible of the conversion of pyruvate into lactate, induced by Ptac2S. This hypothesis could be also confirmed at 24 h. In fact, in Ptac2S treated samples at 24 h, the levels of pyruvate and lactate were comparable and lower, respectively. On the other hand, at 24 h, in cisplatin with respect to both controls and Ptac2S treated samples, pyruvate and lactate decreased. This is of particular importance in light of the fact that many tumors are characterized by an increased expression of the LDHA gene. Indeed, LDH-5 could be considered a target of anti-cancer therapy since its inhibition may reduce the invasive and metastatic potential of tumor cells by decreasing their proliferation ability and reversing their resistance to chemotherapy [60].

SKOV-3 cells metabolic response to the Ptac2S exposure, in comparison with both controls and cisplatin, also resulted in a fast (at 6 h) down-regulation of myo-inositol, PC, and taurine, together with an increase of glycerol. These latter variations were also observed for some other antitumor drugs and could be associated to cell membrane alterations [61]. These data strongly suggest that Ptac2S could cause the cell membrane alteration already at 6 h, differently from cisplatin which gave increase of myo-inositol, PC, and taurine together with an increase of glycerol only after 24 h.

It is also interesting to note that, at short times (6 h), in Ptac2S with respect to cisplatin treated and controls cells, the PC and Cho (choline) levels were lower and equal respectively. In Ptac2S treated cells, the Cho concentration remained constant from 6 to 24 h, while PC decreased with the time. Differently, cisplatin treatment caused the decrease of both PC and Cho, only after 24 h. The Cho decrease has been often associated to apoptosis induction phenomena, as reported for the interaction of various cancer cell types with different drugs [13,62,63].

Cho is known to contribute to the phosphatidylcholine (PTC) biosynthesis, the major component of biological membrane [64]. The drugs inducted apoptosis is generally accompanied by inhibition of the PTC biosynthesis as revealed by the contemporary decrease of Cho and PC [65]. In Ptac2S treated samples, the decrease of PC and the invariant levels of Cho (up to 24 h) could be attributed to an enhanced PC catabolism to choline and/or to an inhibition of PC synthesis, as reported in literature [66]. All these results are in accord with very different targeting of Ptac2S with respect to cisplatin in SKOV-3 cell line and are consistent with previous work aimed to identify, by cellular physiology approaches, the apoptotic pathway induced by this specific Pt drug in several cancer cell lines [27,28,30].

#### 2.2.2. Metabolomic Studies of Lipidic Skov-3 Cells Extracts.

**Figure 7:**
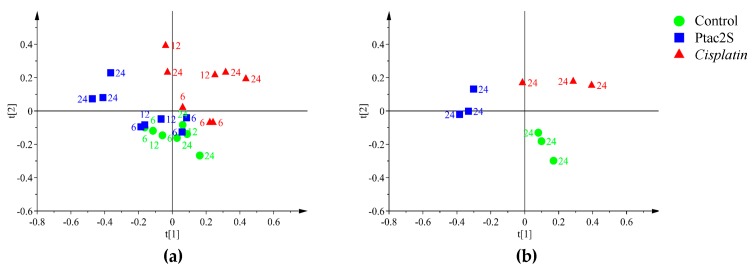
PCA score scatter plots of the 600 MHz ^1^H-ZG-NMR spectra (Pareto scaled) obtained from lipophilic extracts of Ptac2S and *cisplatin* treated SKOV-3 cells in comparison with the untreated control samples. The indicated labels are referred to the culture times. (**a**) PCA t[1]/t[2] (four components give R^2^X = 0.905, Q^2^ = 0.82). (**b**) PCA t[1]/t[2] (two components give R^2^X = 0.854, Q^2^ = 0.771).

Lipids are the main components of the cell membranes and many human diseases, including cancer, are characterized by an alteration of the lipid metabolism [67]. In this context, in order to investigate the possible variations of the lipidic fraction induced by different cell treatments, a further PCA analysis of ^1^H-NMR data of lipidic extracts was conducted. A four-component PCA described 90.5% of the total variance resulting in a good model and class separation among the three considered conditions (Ptac2S and cisplatin treated samples and controls at 6, 12, 24 h). The PCA score plot indicated a different time response also for the lipidic fraction (Figure 7a). In detail, at 24 h after treatment, a marked separation among classes (Ptac2S and cisplatin treated samples and controls) was observed by PCA along the t[1] (Ptac2S with respect to controls and cisplatin) and the t[2] (controls with respect to cisplatin) components, Figure 7b.

The evident differences in metabolites’ expression, obtained at 24 h, under the three condition of treatment (Ptac2S and cisplatin treated samples and controls), were further enhanced by supervised methods (OPLS-DA).

**Figure 8:**
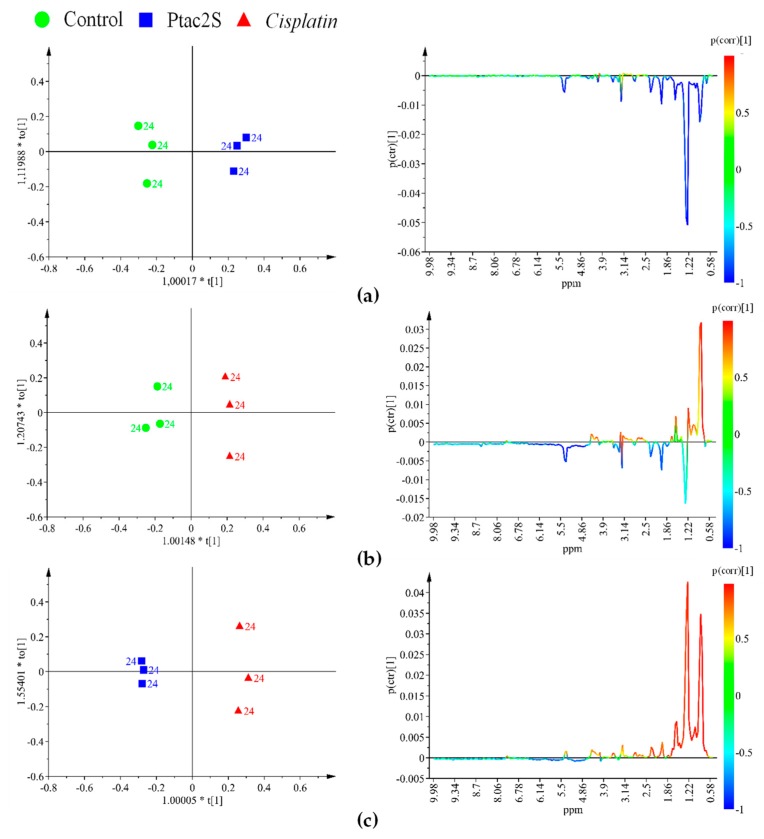
OPLS-DA score plot (left panel) and corresponding coefficient plot (right panel) of the 600 MHz ^1^H-ZG-NMR spectra (Pareto scaled) of SKOV-3 cells lipophilic extracts obtained from different pairwise groups (controls, Ptac2S, and cisplatin) at 24 h. (**a**) OPLS-DA t[1]/t[2] scores scatter plot (two components, 1 predictive + 1 orthogonal give R^2^X = 0.887, R^2^Y = 0.986, Q^2^ = 0.944. (**b**) OPLS-DA t[1]/t[2] scores scatter plot (two components, 1 predictive +1 orthogonal give R^2^X = 0.784, R^2^Y = 0.985, Q^2^ = 0.936). (**c**) OPLS-DA t[1]/t[2] scores scatter plot (two components, 1 predictive + 1 orthogonal give R^2^X = 0.893, R^2^Y = 0.996, Q^2^ = 0.932).

The inspection of the pairwise OPLS-DA models for the different groups (controls, Ptac2S, cisplatin) revealed the metabolites responsible for the observed class separation (Figure 8). In particular, in Ptac2S treated cells, a lower overall lipid level, with respect to controls and cisplatin treated cells, was observed (Table 2). On the other hand, the exposure of SKOV-3 cells to cisplatin, resulted in a lipids increase (particularly cholesterol and triglycerides, TG) with respect to the controls. Furthermore, the comparison between the different treated samples (either with Ptac2S or cisplatin) showed a specific increase of unsaturated lipids in cisplatin with respect to Ptac2S. Such an increase of unsaturated lipids is generally observed as a consequence of the treatment with antitumor drugs of several cancer cell lines [19,21,22,68]. This condition was proposed as a consequence of the membrane degeneration in apoptotic processes, followed by the release of cell membrane fragments as lipid droplets (LD, a neutral core of triglycerides and cholesterol esters surrounded by a monolayer of phospholipids) characterized by high NMR visibility. Indeed, the integral membrane lipids, due to restricted molecular motion, generally give poor NMR signals [19,69]. The accumulation of intracellular LDs reported in literature is strictly related with important cellular processes as proliferation, apoptosis, and necrosis [20,21,64,70].

Cisplatin treatment of cancer cell lines is well known to cause apoptosis, producing a preliminary increase of the cell membrane permeability with consequent enhanced TG and cholesterol levels [20,21,22]. Compared with cisplatin, Ptac2S induces different lipid expression in SKOV-3 cells. In particular, the decrease of both TG and cholesterol (Ptac2S at 24 h), supports the hypothesis of either a decreased biosynthesis or an increased consumption of lipids but also a mechanism of death probably different from apoptosis.

### 2.3. Metabolic Changes of Skov-3 Cells Culture Media Due to Treatment with Ptac2s

The metabolite composition alteration in the culture media allows detection of the utilization and/or release of specific substances, giving also useful information about the physiological status of studied cell cultures [55]. Culture media of treated (Ptac2S or cisplatin) and control cells at the considered time of exposure (6, 12 and 24 h) were collected and analyzed ^1^H-CPMG-NMR, providing the basic information for multivariate data analysis.

Nutrient substrates, such as various amino acids (glutamine, valine, isoleucine, leucine, tyrosine, and phenylalanine) and glucose, constituting the essential elements for cell growth, are evidenced in the NMR spectra of analyzed culture media (Table 1).

**Figure 9:**
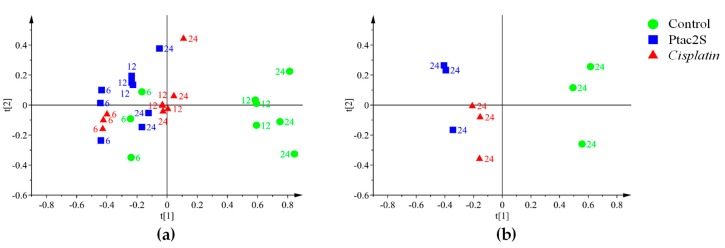
PCA score scatter plot of the 600 MHz ^1^H-CPMG-NMR spectra (Pareto scaled) obtained from recovered culture media of Ptac2S and *cisplatin* treated SKOV-3 cells in comparison with the untreated control samples. The indicated labels are referred to the culture times. (**a**) PCA t[1]/t[2] (four components give R^2^X = 0.969, Q^2^ = 0.936). (**b**) PCA t[1]/t[2] (two components give R^2^X = 0.857, Q^2^ = 0.678).

In the ^1^H-CPMG-NMR based culture media MVA the simple PCA (4 components) explained 96.9% of the total variance and revealed time-dependent responses, along the t[1] component (Figure 9a). Focusing on the 24 h samples a clear discrimination for the composition of SKOV-3 cells culture media of treated (Ptac2S and cisplatin), with respect to controls, was observed by PCA (2 components, 85.7% of the total variance, Figure 9b).

**Figure 10:**
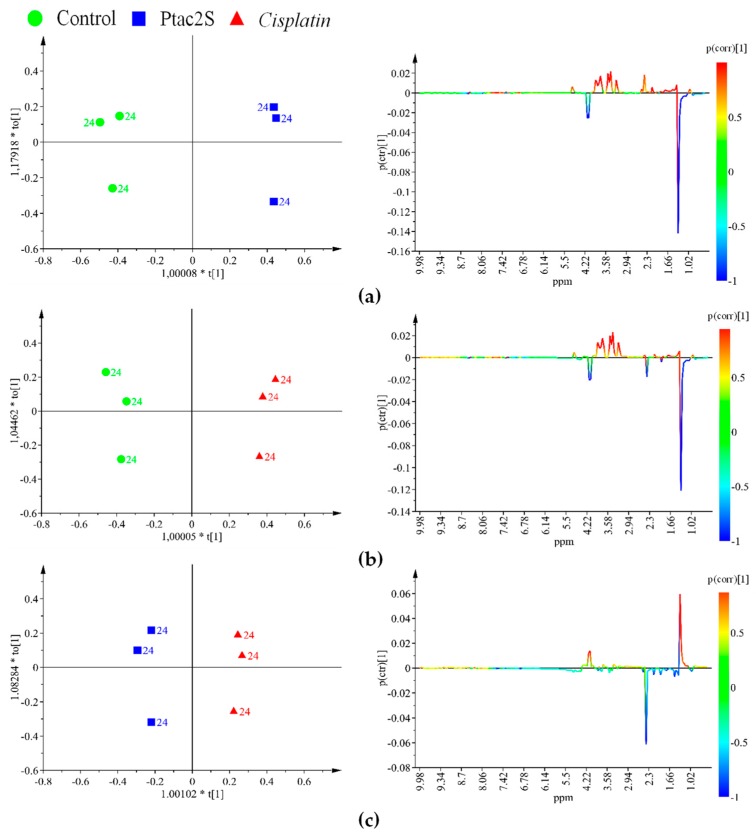
OPLS-DA score scatter plot (left panel) and corresponding coefficient plot (right panel) of the 600 MHz ^1^H-CPMG-NMR spectra (Pareto scaled) of SKOV-3 cells culture media obtained from different pairwise groups (controls, Ptac2S, and cisplatin) at 24 h. (**a**) OPLS-DA t[1]/t[2] (two components, 1 predictive + 1 orthogonal, give R^2^X = 0.944, R^2^Y = 0.995, Q^2^ = 0.99. (**b**) OPLS-DA t[1]/t[2] (two components, 1 predictive + 1 orthogonal, give R^2^X = 0.913, R^2^Y = 0.989, Q^2^ = 0.957). (**c**) OPLS-DA t[1]/t[2] (two components, 1 predictive + 1 orthogonal, give R^2^X = 0.792, R^2^Y = 0.988, Q^2^ = 0.871).

The discrimination between the three groups of samples (controls, Ptac2S, and cisplatin) at 24 h was further enhanced by the OPLS-DA supervised method. Three groups were analyzed pairwise to give three OPLS-DA models (Figure 10). The model’s parameters accounted for clear metabolic differences between controls and Ptac2S or cisplatin treated cells, while less pronounced discrimination was observed between Ptac2S and cisplatin treatment. In detail, in Ptac2S and cisplatin treated samples, higher levels of glutamine and glucose and lower values of lactate were observed with respect to the control samples. In both Ptac2S and cisplatin treated samples, a decrease of the consumption of nutrients (glucose and glutamine) was observed, probably indicating a general slowing down of the SKOV-3 cells’ metabolism, induced by both drugs. Indeed, glutamine is very important to maintain their Krebs cycle efficient in cancer cells [5] and the reduced utilization of glucose and glutamine is often associated to cell death [5,59]. On the other hand, in Ptac2S, the levels of acetate and pyruvate were higher with respect to both controls and cisplatin treated samples suggesting also a specific lactate dehydrogenase inhibition due to Ptac2S.

**Figure 11:**
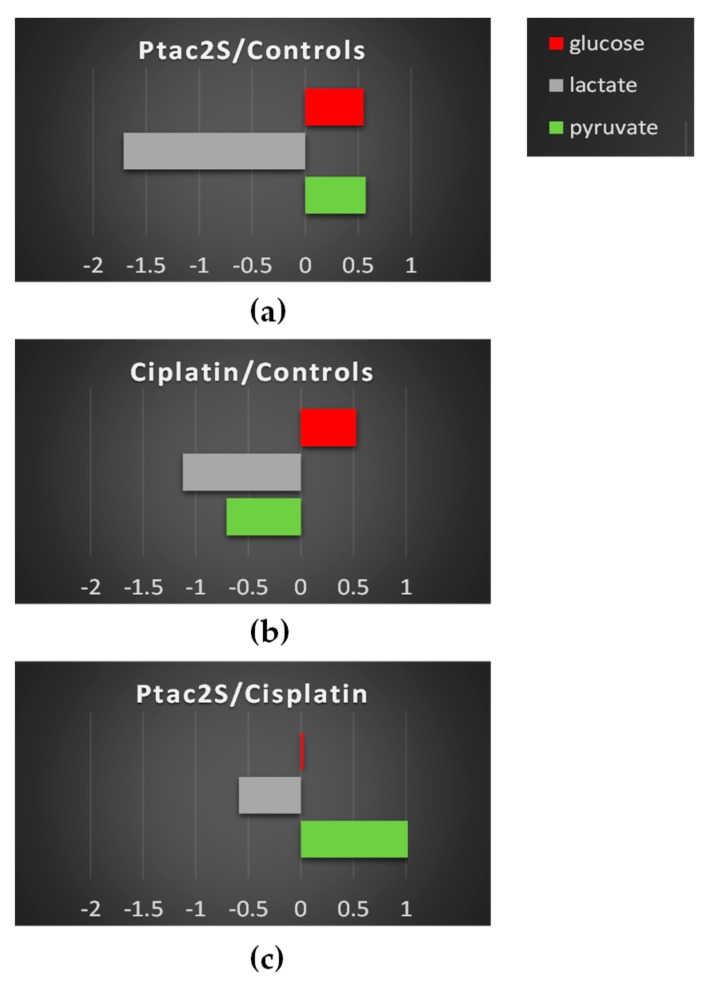
Relevant discriminant metabolites comparison of the culture media of treated, with either Ptac2S, *cisplatin*, and untreated (controls) SKOV-3 cells. The values of −Log_2_(FC) and the *p*-values < 0.05 are provided (Student’s *t*-test). (**a**–**b**) Metabolites with −Log_2_ (FC) negative values have a lower concentration compared to those of the 24 h control, while −Log_2_ (FC) positive values have higher concentration compared to those of the 24 h control. (**c**) Metabolites with −Log_2_ (FC) negative values have a lower concentration compared to those of the 24 h *cisplatin* group, while −Log_2_ (FC) positive values have a higher concentration compared to those of the 24 h *cisplatin* group.

As it is well known, glucose enters the cells by using its specific transporter. Once in the cell, it is converted to pyruvate through glycolysis [3,4,5]. Pyruvate is converted either to lactate (which is excreted in the media) by lactate dehydrogenase or to Acetyl Co-A (which enters the Krebs cycle) by pyruvate dehydrogenase [71]. The tendency to convert pyruvate into lactate, known as the “Warburg effect”, is frequently observed in cancer cells [3,4,5]. In fact, in controls, with respect to cisplatin treated samples, higher levels of lactate and pyruvate were consistently found. Interestingly, in Ptac2S treated cells, lower levels of lactate and higher levels of pyruvate were observed, with respect to both controls and cisplatin treated samples (Figure 11). This trend is consistent with the above discussed results of cell lysate aqueous extract analysis and suggests the inhibition of conversion of pyruvate into lactate due to Ptac2S. Quantitative data related to relevant discriminating metabolites observed in OPLS-DA models were obtained with a targeted analysis by ^1^H-NMR spectra integration. Fold change (FC) ratios (log2) of the normalized median intensity for the distinctive signals in the spectra were calculated and are reported in Figure 11.

## 3. Materials and Methods

### 3.1. Synthesis of Complexes

All solvents and reagents were purchased from Aldrich Chemical Company and used as received, except otherwise stated. Cisplatin, *cis*-[PtCl_2_(NH_3_)_2_], and Ptac2S, [Pt(*O,O′*-acac)(γ-acac)(DMS)], were prepared according to previously reported procedures [28,72].

### 3.2. SKOV-3 Cell Cultures and Drugs Administration

The human ovarian SKOV-3 cancer cells were purchased from the Merck KGaA, Darmstadt, Germany. These were cultured in DulbeccO′s High Glucose Modified Eagle’s Medium (DMEM) supplemented with 10% fetal bovine serum, 100 U/mL penicillin, and 100 µg/mL streptomycin at 37 °C under an atmosphere of 5% CO_2_ in air.

#### 3.2.1. Cytotoxicity Assay

The cytotoxicity assay was performed to evaluate the IC_50_ (half-maximal inhibitor concentration) of Ptac2S and cisplatin in SKOV-3 cells. One hundred μL of a SKOV-3 cells suspension were seeded into each well of a 96-well culture plate at concentration of 6 × 10^4^ cells/mL in complete medium. After overnight incubation, cells were treated with variable Ptac2S and cisplatin concentrations (1, 5, 10, 25, 50, 100 μM) for 12, 24, and 48 h time intervals. Afterwards, 10 μL of 3-[4,5-dimethylthazol-2-yl]-2,5-diphenyl tetrazolium bromide (MTT), purchased from Sigma-Aldrich (Darmstadt, Germany), were added in each well and incubated for additional 4 h, with a final MTT concentration into each well-plate of 0.5 mg/mL. Living cells reduce the yellow MTT to the purple formazan crystals. After removing the supernatant fraction, cells were incubated with 100 μL of isopropanol while shaking for 5–10 min. This method measures the reduction of MTT by active mitochondria, which results in intensity of the colored product as quantified by a spectrophotometer at 550 nm. The intensity of the colored product formed is directly proportional to the number of living cells present in a sample. The absorbance of the control cells was taken as 100% viability, and the values of treated cells were calculated as a percentage compared to control. Experiments were performed in triplicate. The half-maximal inhibitor concentration (IC_50_) of Ptac2S and cisplatin, at 24 h, was calculated and used to prepare the metabolomic assay.

#### 3.2.2. Metabolomic Assay.

SKOV-3 cells for metabolomics analyses were cultured in T75 flasks to a confluence of 70% (ca 106 cells/flask). The next day, fresh culture medium supplemented with 6.2 ± 2.8 µM of Ptac2S corresponding to the half maximal inhibitor concentration (IC_50_) at 24 h (drug concentration causing 50% of cell death) and with 87.5 ± 3.5 µM cisplatin (IC_50_) at 24 h, respectively. For control cells, fresh medium without drug was added. Cells were then incubated for 6, 12, and 24 h, harvested by tripsinization, washed with PBS, and pelleted by centrifugation (1000 rpm × 10 min). The same procedure was rigorously used for all samples to minimize experimental variability. For each time point, three independent assays were performed.

NMR samples were prepared from pelleted SKOV-3 cells and from each respective recovered culture medium. The pelleted SKOV-3 cells were extracted according to a previously reported procedure, with a methanol/chloroform/water mixture, which was then treated further in order to separate polar and lipophilic fractions [73].

The polar fractions were resuspended in 580 µL buffer (0.1 M K_2_HPO_4_, pH 7.4 in D_2_O, 0.2 mM TSP, and 2 mM sodium azide), to minimize variations in metabolite NMR chemical shifts. Samples were centrifuged at 12,000 g for 5 min at 4 °C to remove any solid debris and 550 µL of the supernatant transfer in a 5 mm NMR tube.

The lipophilic extracts were resuspended in 580 µL of deuterated solvent (2:1 mixture of CDCl_3_ containing 0.03 *v*/*v* TMS, and CD_3_OD) then vortexed and centrifuged. The supernatants (550 µL) were finally transferred in 5 mm NMR tubes.

Each of the culture media sample, (900 µL) was added 100 µL buffer (1.5M K_2_HPO_4_, 2 mM TSP, and 2 mM sodium azide), to minimize variations in metabolite NMR chemical shifts. Samples were vortexed and centrifuged. The supernatants (600 µL) were finally placed in 5 mm outer diameter NMR tubes.

Spectra were acquired for controls, Ptac2S, and cisplatin-treated (at 6, 12, and 24 h) SKOV-3 cells, at different time intervals for culture media, aqueous, and lipophilic extracts.

### 3.3. NMR Measurements

All measurements were performed on a Bruker Avance III 600 Ascend NMR spectrometer (Bruker, Karlsruhe, Germany) operating at 600.13 MHz for ^1^H observation, equipped with a z axis gradient coil and automatic tuning-matching (ATM). A time delay of 5 min was set between sample injection and pre-acquisition calibrations to ensure complete temperature equilibration (300 K). Experiments were run at 300 K in automation mode after loading individual samples on a Bruker Automatic Sample Changer, interfaced with the software Icon NMR (Bruker).

For each aqueous extract and culture medium sample, a one-dimensional ZGPR and CPMG experiment with a transverse-relaxation-filter incorporating pulse sequence (referred to as Carr–Purcell–Meiboom–Gill spin-echo sequence, CMPG) was run with 128 scans, a total spin–spin relaxation delay of 20 µs, and solvent signal saturation during the relaxation delay. The FIDs were multiplied by an exponential weighting function corresponding to a line broadening of 0.3 Hz before Fourier transformation, phasing, and base line correction. All spectra were referenced to the trimethylsilyl propionate (TSP) signal (δ = 0.00 ppm). For each lipid extract, a one-dimensional experiment (ZG experiment) was run with 16 scans, 64 K time domain, spectral width 20.0276 ppm (12019.230 Hz), p1 10 µs. All spectra were referenced to the tetramethylsilane (TMS) signal (δ = 0.00 ppm). The metabolites were assigned on the basis of 2D-NMR spectra analysis (^1^H-COSY, ^1^H-^13^C HSQC and HMBC) and comparison with Human Metabolome Database and other published data [74].

### 3.4. Spectral Processing and Multivariate Data Analysis

^1^H-NMR spectra were automatically divided in rectangular buckets of fixed 0.04 ppm width and integrated using Bruker Amix 3.9.13 (Bruker, Biospin) software. For aqueous intracellular extracts and culture media, the spectral region between 4.5 and 5 ppm (residual protic water signal) was discarded to exclude the effects of variability in water suppression signal. The remaining buckets in the range of 10.00–0.50 ppm were normalized to total area to minimize small differences and subsequently mean-centered. For lipid extracts, to exclude signals of the residuals non-deuterated chloroform and its carbon satellites and the residual methanol, the spectral regions between 7.60–6.90 and 3.60–3.00 ppm, respectively, were excluded and the remaining buckets in the range of 10.0–0.5 ppm were normalized to the total area to minimize small differences and subsequently mean-centered. The Pareto scaling method, which is performed by dividing the mean-centered data by the square root of the standard deviation, was then applied to the variables. Multivariate statistical analyses were performed with the software SIMCA-P (V14, Umetrics, Umea, Sweden). Principal component analysis (PCA), an unsupervised pattern recognition method, was performed in order to identify general metabolic trends and possible outliers. Then, orthogonal projections to latent structure discriminant analysis (OPLS-DA), a supervised multivariate data analysis, was carried out to improve the statistically significant metabolite variations related to complex exposures.

The statistical models’ (PCA and OPLS-DA) performance was evaluated by R^2^(cum) and Q^2^(cum) parameters for goodness of fit and prediction, respectively, according to seven-fold internal cross-validation further evaluated with permutation test (100 permutations) of SIMCA-P software [75,76,77].

## 4. Conclusions

**Figure 12:**
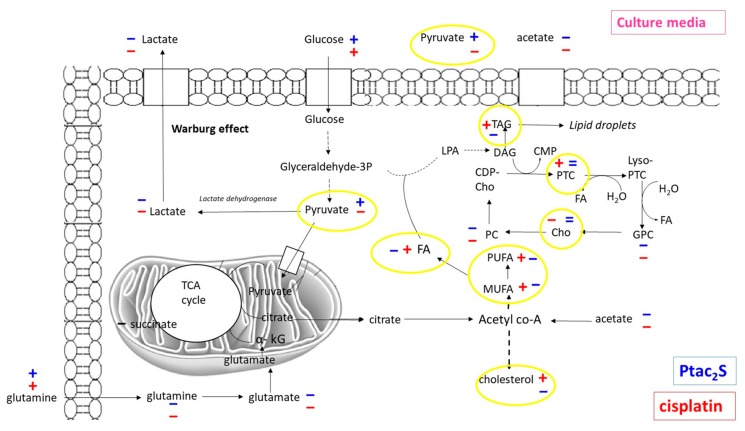
Schematic representation of main metabolic pathways altered at 24 h after Ptac2S (blue) and *cisplatin* (red) treatment of SKOV-3 cells. Comparison with the 24 h of the controls. Positive (+) and negative (−) signs indicate positive and negative correlations in the concentration, respectively.

Based on MVA (multivariate data analysis) results, the effects of Ptac2S and cisplatin on the SKOV-3 cells metabolome were shown to be significantly distinct. While Ptac2S treated cells were characterized at short times (6 h) by the decrease in Krebs’ cycle efficiency (decrease of succinate, glutamine, glutamate, lactate levels), and the cell membrane alteration (decrease of myo-inositol, PC and taurine together with an increase of glycerol), cisplatin was found to induce the same cytotoxic effects only at long time (24 h). Pyruvate (the end-product of glycolysis) seemed also to play a key role in the Ptac2S mechanism of action, as an indicator of an unbalanced conversion of pyruvate into lactate (Warburg effect). Indeed, at 24 h, in Ptac2S treated cells, with respect to controls (in both aqueous extracts and culture media), higher levels of pyruvate and lower levels of lactate were found (Figure 12). On the other hand, for cisplatin treated cells (in both aqueous extracts and culture media), lower levels of pyruvate and lactate were found with respect to the controls. This insight suggests that in Ptac2S treated cells, differently from cisplatin, the inhibition of lactate dehydrogenase, the enzyme responsible for the pyruvate conversion into lactate may also occur. Further work specifically devoted to studying the relevant enzymatic reactions analogously to that already performed for this novel complex with other enzymes could confirm this metabolic profiling derived hypothesis [78].

Furthermore, at 24 h, in MVA of lipid extracts of Ptac2S treated cells, a lower overall lipid levels (TG, cholesterol, Unsaturated Fatty Acids), with respect to both controls and cisplatin treated, was observed. The lipid profile of cisplatin treated and controls cells revealed the typical behavior of cells undergoing apoptosis and/or proliferation [20,21,22,64]. Differently, the lower overall lipids levels observed in Ptac2S treated cells clearly indicated decreased lipid biosynthesis or increased consumption, but also a mechanism of death probably different from apoptosis [20,21,22,70].

In conclusion, the proposed NMR-based metabolomic analysis confirms that Ptac2S limits cancer cell proliferation, with a mechanism very different from that of cisplatin, which is essentially cytoplasmic, in accordance with previous studies [26,27,28,29,30,31].

## Figures and Tables

**Table 1:** Relevant metabolites derived from the pairwise OPLS-DA models of ^1^H-NMR analysis of aqueous extracts between controls and Ptac2S and cisplatin treated samples. Positive (+) and negative (−) sign indicate positive and negative correlation in the concentration, respectively.

Metabolites	δ(^1^H)/ppm	Ptac2S/Controls		Cisplatin/Controls		Ptac2S/Cisplatin	
		6 h	24 h	6 h	24 h	6 h	24 h
Acetate	1.94(s)	−	−	−	−		+
Alanine	1.48(d); 3.80(m)	−	−			−	
AMP	8.23(s); 8.56(s)	−	−		−	−	
Cho	3.21(s)	+			−		+
Creatine	3.05(s); 3.93(s)	−	−	+	−	−	+
Dimethylamine	2.74(s)	−	+	−	+	−	+
Formate	8.46				−	−	
GPC	3.24(s)				−		
Glycerol	3.65(m)		+		+	+	+
Glycine	3.56(s)	−	−	−	−	−	
Glutamine	2.15(m); 2.45(m); 3.77(m)	−	−	−	−	−	−
Glutamate	2.05(m); 2.12(m); 2.35(m)	−	−	−	−	−	−
Lactate	1.32(d); 4.11(q)	−	−	−	−	+	+
*m*-inositol	3.25(t); 3.54(dd); 3.62(t); 4.05(t)		−		−	−	
Isoleucine	0.94(t); 1.01(d); 1.98(m)	−		−		+	
Leucine	0.96(d); 0.97(d); 1.72(m); 1.73 (m)	−				+	
PC	3.23(s)	−	−	+	−	−	+
Pyruvate	2.38(s)				−		+
Serine	3.84(dd); 3.95(dd); 3.98(dd)	+	+		+	+	
Succinate	2.41(s)	−	−		−	−	+
Taurine	3.25(t); 3.43(t)	−	−		−	−	−
TMAO	2.9(s)				+		
UDP-Glucose	5.97(d); 5.61(m); 6.10(d); 7.93(d)				−	−	
Valine	1.00(d); 1.05(d); 2.28(m); 3.62(d)	−				+	

**Table 2:** Relevant metabolites derived from the pairwise OPLS-DA models of ^1^H-NMR analysis of lipidic extracts between controls and Ptac2S and cisplatin treated samples at 24 h of treatment. Positive (+) and negative (−) signs indicate positive and negative correlation in the concentration, respectively.

Metabolites	δ(^1^H)/ppm	Ptac2S/Controls	Cisplatin/Controls	Ptac2S/Cisplatin
Cholesterol CH_3_-18	1.94(s)	−	−	
Cholesterol CH_3_-26,27	1.48(d); 3.80(m)	−		−
CH_3_ of all FAs (MUFA and DUFA) except n-3	8.23(s); 8.56(s)	−		−
Cholesterol CH_3_-21	3.21(s)	+		
all PUFAs n-3	3.05(s); 3.93(s)	−	+	−
Cholesterol CH_3_-19	2,74(s)	−	−	−
CH_2_ of all fatty chain	8.46			−
Cholesterol CH_2_	3.24(s)			
CH_2_CH_2_COOH of all fatty acids	3.65(m)			+
CH_2_CH=CH of MUFAs, DUFAs and PUFAs	3.56(s)	−	−	−
CH_2_CH=CH of all PUFAs n-3	2.15(m); 2.45(m); 3.77(m)	−	−	−
CH_2_COOH of all FAs	2.05(m); 2.12(m); 2.35(m)	−	−	−
CH=CH-CH_2_-CH=CH of DUFA and PUFA	1.32(d); 4.11(q)	−	−	+
Phosphatidylcholine N-(CH_3_)_3_	3.25(t); 3.54(dd); 3.62 t); 4.05(t)			−
Phosphatidylcholine CH_2_N	0.94(t); 1.01(d); 1.98(m)	−	−	+
Glycerophospholipid backbone 3-CH_2_	0.96(d); 0.97(d); 1.72(m); 1.73(m)	−		+
Glycerol backbone of TG 1,3-CH_2_	3.23(s)	−	+	−
Phosphatidylcholine CH_2_OP	2.38(s)			
Glycerol backbone of TG 2-CH	3.84(dd); 3.95(dd); 3.98(dd)	+		+
CH=CH of all MUFA, DUFA, PUFA	2.41(s)	−		−
